# Efficient phenol degradation by laccase immobilized on functional magnetic nanoparticles in fixed bed reactor under high‐gradient magnetic field

**DOI:** 10.1002/elsc.202100009

**Published:** 2021-05-06

**Authors:** Ting‐Ting Xia, Mei Feng, Chun‐Lei Liu, Chun‐Zhao Liu, Chen Guo

**Affiliations:** ^1^ State Key Laboratory of Biochemical Engineering & Key Laboratory of Green Process and Engineering Institute of Process Engineering Chinese Academy of Sciences Beijing P. R. China; ^2^ University of Chinese Academy of Sciences Beijing P. R. China; ^3^ Key Laboratory of Radiological Protection and Nuclear Emergency China CDC National Institute for Radiological Protection Chinese Center for Disease Control and Prevention Beijing P. R. China; ^4^ State Key Laboratory of Bio‐fibers and Eco‐textiles Institute of Biochemical Engineering Affiliated Qingdao Central Hospital College of Materials Science and Engineering Qingdao University Qingdao P. R. China

**Keywords:** fixed bed reactor, high‐gradient magnetic field, magnetic immobilized laccase, phenol degradation

## Abstract

Enzymatic degradation of emerging contaminants has gained great interest for the past few years. However, free enzyme often incurs high costs in practice. The immobilized laccase on the polyethylenimine (PEI)‐functionalized magnetic nanoparticles (Fe_3_O_4_–NH_2_–PEI–laccase) was fabricated to efficiently degrade phenolic compounds continuously in a newly fixed bed reactor under a high‐gradient magnetic field. The degradation rate of continuous treatment in the bed after 18 h was 2.38 times as high as that of batch treatment after six successive operations with the same treatment duration. Under the optimal conditions of volume fraction of nickel wires mesh, flow rate of phenol solution, phenol concentration, and Fe_3_O_4_–NH_2_–PEI–laccase amount, the degradation rate of phenol kept over 70.30% in 48 h continuous treatment. The fixed bed reactor filled with Fe_3_O_4_–NH_2_–PEI–laccase provided a promising avenue for the continuous biodegradation of phenolic compounds for industrial wastewater in practice.

**Abbreviations**
GA: glutaraldehyde
NPs: nanoparticles
PEI: polyethylenimine
VSM: vibrating sample magnetometer

## 1 INTRODUCTION

Phenolic compounds are troublesome pollutants found in effluents from various industries, such as pulp and paper manufacturing, plastic and resin production, production of pesticides, and pharmaceutical industry [1, 2]. Phenol, with rather high solubility [3], has devastating toxic effect on living organisms even at low concentration [4]. Phenol has been considered by the US Environmental Protection Agency (EPA) as a priority pollutant [5, 6]. Therefore, the removal of phenol in wastewater effectively is a burning environmental and health issue.

Enzymatic catalysis has received great attention to friendly, mildly, and effectively remove phenolic pollutant without creating harsh side effects [7, 8]. Laccase (benzenediol: oxygen oxidoreductases, EC 1.10.3.2) as an ideally “green” multi‐copper oxidase has been widely applied in removing of phenolic pollutants [9, 10]. Up to now, free laccase has been successfully immobilized on various carriers, especially magnetic carriers to improve stability, facilitate products separation, and realize catalyst reuse in many applications [11, 12]. Recently, immobilized enzyme on magnetic carriers was used to improve catalytic performance under alternating magnetic field as well as in the magnetically stabilized fluidized bed reactor [13, 14, 15, 16, 17, 18]. Because the immobilized enzyme was suspended under the magnetic field, the resulted products were discharged continuously. In additional, some fixed bed reactors coupled with permanent magnet that provided static magnetic field or high‐gradient magnetic field were also built for enhance the catalytic active of these immobilized enzymes [19, 20].

The objective of the current study is to immobilize laccase onto amine‐functioned Fe_3_O_4_ nanoparticles modified with polyethylenimine (PEI) (Fe_3_O_4_–NH_2_–PEI–laccase) to efficiently degrade phenolic compounds continuously in a newly fixed bed under high‐gradient magnetic field. In order to test and verify the feasibility and advantage of the designed bed, the difference between continuous and batch treatment was compared and the influences of operational parameters were investigated.

## 2 MATERIALS AND METHODS

### 2.1 Chemicals and reagents

Amine‐functionalized Fe_3_O_4_ nanoparticles (Fe_3_O_4_–NH_2_ NPs) (the size is 15 nm and the concentration of primary amino groups on the surface is 0.20 mmol/g) were obtained from the Beijing GiGNano Biointerface Company. Laccase from *Trametes versicolor* (p‐diphenol: dioxygen oxidoreductases, EC 1.10.3.2) was from our group. PEI (branched, Mn∼1200) was purchased from Sigma‐Aldrich (USA). Sodium chloroacetate was provided by Aladdin (Shanghai). All other chemicals including glutaraldehyde (GA), ethylenediamine, cupric sulfate (CuSO_4_·5H_2_O), and phenol were of analytical grade and acquired from Beijing Chemical Reagents Company (Beijing).

**PRACTICAL APPLICATION**

Enzymatic degradation of emerging contaminants has gained great interest for the past few years. The magnetic laccase catalyst coupled with the fixed bed reactor under high‐gradient magnetic field presented in this study provides a promising platform for emerging organic pollutants biodegradation in practice.

### 2.2 Preparation of PEI functionalized Fe_3_O_4_ nanoparticles for laccase immobilization

According to the methods reported previously [21, 22], the branched PEI (Mn∼1200) was covalently modified on Fe_3_O_4_–NH_2_ NPs using GA as reactive intermediate. Fe_3_O_4_–NH_2_ NPs and the yielded products of Fe_3_O_4_–NH_2_–PEI NPs were both chelated with Cu^2+^ to immobilize laccase separately. The amount of laccase immobilized on the NPs was measured by the Bradford protein assay method [23]. The magnetic properties of Fe_3_O_4_–NH_2_–PEI–laccase were measured with a vibrating sample magnetometer (VSM, Model 4 HF VSM of ADE Technologies, Inc.) at 19°C.

### 2.3 Experimental apparatus of a fixed bed reactor under high‐gradient magnetic field

**SCHEME 1:**
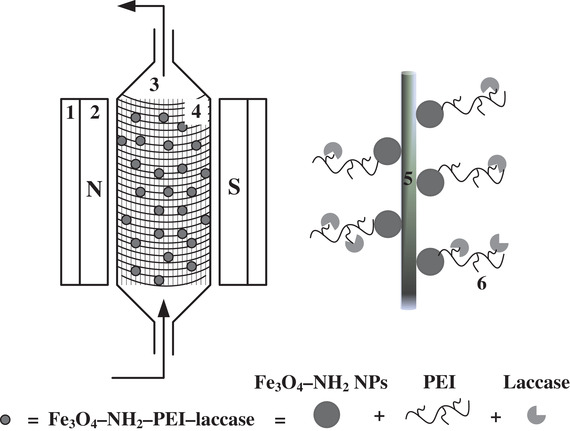
The experimental apparatus of the fixed bed using high‐gradient magnetic separation. (1) Low carbon steel yoke, (2) permanent magnet, (3) separation column, (4) ferromagnetic nickel wires mesh, (5) nickel wire, and (6) Fe_3_O_4_–NH_2_–PEI–laccase

The reactor was consisted of magnetic circuit, separation column, and solution delivery system (Scheme 1). The magnetic circuit included U‐Shaped low carbon steel yoke and two parallel rectangular Nd—Fe—B permanent magnets. The magnetic intensity (*H*o) was 2800 Gs. The separation column was in the center of the axial magnetic field, which was made up of polymethyl methacrylate with a diameter of 10 mm and a height of 60 mm. Different length of nickel wires mesh with a wire diameter of 1 mm and a mesh width of 58 mm was rolled into a cylinder to be filled into the separation column. Ferromagnetic wires mesh was magnetized by the externally applied magnetic field. High‐gradient magnetic fields were produced around the wires, facilitating the stable capture of magnetic immobilized laccase from fluid suspensions. The solution delivery system connected with the known concentration phenol solution, separation column, and degraded phenol solution by peristaltic pump and rubber tube.

### 2.4 Continuous degradation of phenol in the fixed bed reactor

As shown in Scheme 1, the magnetic immobilized laccase dispersed well in the NaAC‐HAC buffer (pH = 4) was pumped into the separation column filled with nickel wires mesh from bottom inlet without applying the magnetic field. When the column was full, it was put into the magnetic field. The bottom inlet of the column was opened to remove the buffer. The phenol concentration was analyzed by measurement the absorbance at a wavelength of 510 nm using UV‐2100 spectrophotometer after color development by 4‐aminoantipyrene method [24]. The phenol degradation rate was calculated according to the following equation (1).

(1) $$\begin{array}{ccc} & & \text{Phenol}\;\text{degradation}\;\text{rate}\;(\%)\\ & & \;=\left(1-\frac{\text{Phenol}\;\text{concentration}\;\text{in}\;\text{the}\;\text{effluent}}{\text{Initial}\;\text{phenol}\;\text{concentration}}\right)\times 100\% \end{array}$$

10 mg of Fe_3_O_4_—NH_2_—PEI–laccase was uniformly absorbed in the separation column filled with 6.6 g of nickel wires to degrade phenol solution (dissolved in NaAc‐HAc buffer [pH = 5.5]) at the flow rate of 25 μL/min. The equilibrium time of phenol degradation in the fixed bed was obtained. In the subsequent experiments, the sample was collected from upper outlet after the equilibrium time, and the equilibrium time acted as interval time.

The full volume of phenol solution in the separation column was 4.5 mL. The dynamic curve of phenol degradation by Fe_3_O_4_—NH_2_—PEI—laccase with mechanical stirring of 150 rpm (10 mg of Fe_3_O_4_—NH_2_—PEI—laccase, 4.5 mL of phenol solution [50 μg/mL]) (data not shown) was measured. The time required in every batch treatment of phenol was determined according to the equilibrium time. After every batch treatment, the Fe_3_O_4_—NH_2_—PEI—laccase was separated using magnet and washed with NaAc‐HAc buffer for reuse.

In order to investigate the effect of PEI on the phenol degradation rate, 10 mg of Fe_3_O_4_–NH_2_–laccase and Fe_3_O_4_—NH_2_—PEI—laccase was separately pumped into the separation column filled with nickel wires mesh (6.6 g) to degrade phenol (50 μg/mL) at the flow rate of 25 μL/min. Then the more suitable immobilized laccase was selected to study the effects of the volume fractions of nickel wires mesh in separation column, the flow rate of phenol solution, phenol concentration, and immobilized laccase amount. The research of continuous degradation for a long time was carried out under the most appropriate conditions.

## 3 RESULTS AND DISCUSSION

### 3.1 Magnetic property of Fe_3_O_4_—NH_2_—PEI—laccase

**FIGURE 1:**
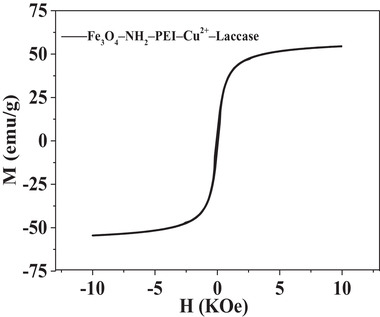
Magnetic hysteresis loops of the Fe_3_O_4_—NH_2_—PEI—laccase

Magnetic particles with large magnetic susceptibility are essential in the fixed bed under high‐gradient magnetic field [25, 26, 27]. The magnetization curve applied magnetic field (M—H loops) of Fe_3_O_4_—NH_2_—PEI—laccase was shown in Figure 1. Fe_3_O_4_—NH_2_—PEI—laccase behaved superparamagnetically and the saturated magnetization is 54.5 emu/g, which was higher than magnetic fluids and other magnetic nanoparticles used in magnetically fixed bed [19]. Therefore, the saturated magnetization was enough to separate the particles from the solution and avoid immobilized laccase leaching from the separation column.

### 3.2 Compare the degradation rate of phenol by the immobilized laccase between the fixed bed and the shake flask

**FIGURE 2:**
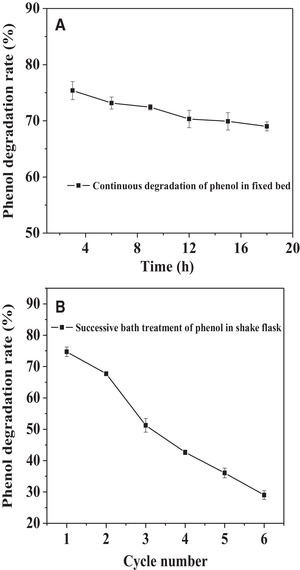
Continuous degradation of phenol by Fe_3_O_4_—NH_2_—PEI—laccase in the fixed bed reactor (A) and the same amount of Fe_3_O_4_—NH_2_—PEI—laccase reused in the shake flask (B)

The equilibrium time of phenol degradation in the fixed bed and in the shake flask was the same: 3 h (Figure S1). 10 mg of Fe_3_O_4_—NH_2_—PEI—laccase was applied to continuously degrade phenol in the fixed bed. The sample was collected from upper outlet every 3 h. Meanwhile, the same amount of Fe_3_O_4_—NH_2_—PEI—laccase was reused six times to degrade 4.5 mL of phenol (50 μg/mL) with mechanical stirring of 150 rpm in the flasks. According to the equilibrium time, each reaction time was 3 h. The duration and the total volume (27 mL) of treated phenol was the same as the treatment in the fixed bed. As shown in Figure 2A, the phenol degradation rate decreased gradually from 75.59 to 68.15% after the continuous treatment in the fixed bed for 18 h. It decreased fast from 73.14 to 28.59% after six successive operations in the batch treatment (Figure 2B). The beginning degradation rate was almost the same, while the end degradation rate of continuous treatment was 2.38 times as high as that of batch treatment. The continuous treatment in the fixed bed achieved higher phenol degradation efficiency (Figure 2). In the meantime, the immobilized laccase was absorbed on the nickel wires uniformly and avoids aggregating as in the batch treatment. The oxidation of phenol catalyzed by the immobilized laccase resulted in quinonoid derivatives or homomolecular dimers, the increasing diameter of the above polymers was observed during the phenol oxidation process in our previous study [28]. As a result, these products coexisted with immobilized laccase in the overall batch operation and might adhere onto the immobilized laccase for inhibiting its activity. However, the oxidation products from the reaction solution were efficiently removed to some extent in the continuous treatment process. In addition, these polymers exhibited inhibitory effect on the phenol oxidation by both free and immobilized laccase in our previous study, but the immobilized laccase showed higher resistance to these degradation products in comparison with the free one [28].

**FIGURE 3:**
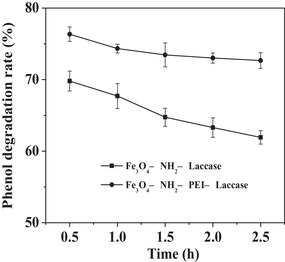
The effect of functionalized PEI on the phenol degradation rate in the fixed bed reactor

Comparison with Fe_3_O_4_—NH_2_—laccase without PEI, the Fe_3_O_4_—NH_2_—PEI—laccase achieved higher phenol degradation rate (Figure 3). After 150 min reaction, the degradation rate catalyzed by Fe_3_O_4_—NH_2_—PEI—laccase stabilized at 72.93 %. The great degradation rate using Fe_3_O_4_—NH_2_—PEI—laccase was due to that the laccase immobilized on the long flexible spacer‐arm functionalized magnetic carrier freely dispersed in high‐gradient magnetic field inside the reactor, which decreased interfacial mass transfer resistance and reduce deposition of degradation products.

### 3.3 Effect of volume fraction of nickel wire mesh on degradation rate in fixed bed reactor

**FIGURE 4:**
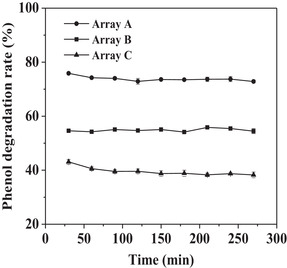
Effect of design of nickel wires mesh in separation column on the phenol degradation rate in the fixed bed reactor. The volume fractions of the wire mesh in the column were 8.1% (Array A), 14.5% (Array B), and 21% (Array C), respectively

Different length of nickel wire mesh was rolled into three different amount of cylinder (3.4 g/6.6 g/9.4 g) to form three different arrays (Array A/B/C). The volume fractions of the wire mesh in the column were 8.1% (A), 14.5% (B), and 21% (C), respectively. 10 mg of Fe_3_O_4_—NH_2_—PEI—laccase was pumped into the three kinds of arrays filled separation column to degrade phenol (50 μg/mL) at the flow rate of 25 μL/min, respectively. As shown in Figure 4, the Array B gave the best degradation rate among all arrays tested. The higher the volume fraction of the wire mesh was, and the more Fe_3_O_4_—NH_2_—PEI—laccase was absorbed on the wires. As a result, the interaction between laccase and phenol was enhanced for efficient degradation. However, too many wires did not provide enough space for molecular chain of PEI to swing freely, thus the immobilized laccase did not disperse freely in the column. In addition, too many wires also blocked the flow channel of phenol solution and product diffusion. The volume fraction of Array C was too high to provide enough porosity for the swinging of PEI and the flow of phenol and the products.

### 3.4 Effect of flow rate of phenol solution and Fe_3_O_4_—NH_2_—PEI—laccase amount on the degradation rate in the fixed bed reactor

**FIGURE 5:**
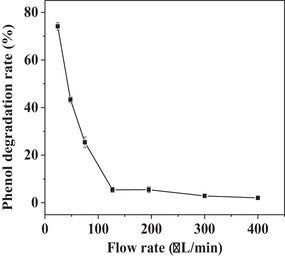
Effect of flow rate on the phenol degradation rate in the fixed bed reactor

As shown in Figure 5, in the flow rate range from 25 to 127 μL/min, the degradation rate decreased quickly from 72.93 to 4.25% because that the over loaded phenol exceeded the catalytic ability of the immobilized laccase in the fixed bed reactor. When the flow rate was high than 127 μL/min, the degradation rate tended to be constant as 4.25% due to the shortened resident time of phenol in the fixed bed reactor. Adequate residence time was needed to ensure that the phenol in the flowing solution was interacting with the immobilized laccase active sites sufficiently for efficient catalysis [29, 30]. When the flow rate was higher, there was less time for phenol to contact with the immobilized laccase for biodegradation. As a result, the degradation rate of phenol was very low in the continuous treatment process.

**FIGURE 6:**
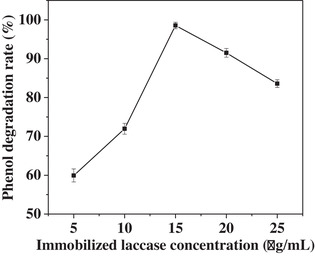
The effect of Fe_3_O_4_—NH_2_—PEI—laccase amount on the degradation rate in the fixed bed reactor

As shown in Figure 6, when the immobilized laccase amount was increased from 5.0 to 15.0 mg, phenol degradation rate was obviously enhanced from 58.29 to 98.33%. However, when the immobilized laccase concentration was higher than 15 mg, the degradation rate decreased from 98.33 to 83.25% due that the overlarge immobilized laccase absorbed on nickel wires blocked the flow channel of phenol and the resulted products.

**FIGURE 7:**
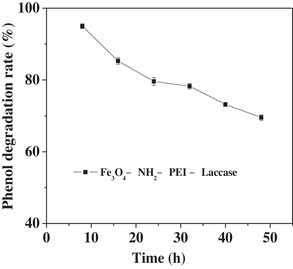
Continuous degradation of phenol by Fe_3_O_4_—NH_2_—PEI—laccase in the fixed bed reactor

Under the above optimal conditions, 15 mg of Fe_3_O_4_—NH_2_—PEI—laccase was absorbed on the arrays B filled separation column to degrade phenol (50 μg/mL) at the flow rate of 25 μL/min. As shown in Figure 7, the degradation rate kept over 70.30% in 48 h under the continuous operation. The decreased degradation rate might be due to inactivation of laccase caused by the unremoved products or the always contacted phenol solution [31]. This was higher than the degradation rate (50%) using packed bed bioreactor after eighth run (48 h) in Niladevi'research, in which enzyme leaching was inevitable [32].

## 4 CONCLUDING REMARKS

A fixed‐bed reaction under high‐gradient magnetic field was established successfully to degrade phenol using immobilized laccase on the PEI‐functionalized magnetic nanoparticles. The phenol degradation rate catalyzed by Fe_3_O_4_—NH_2_—PEI—laccase in magnetically fixed bed reactor was significantly higher than that in the successive batch treatment within the same operation period. Under the optimized operation conditions, the immobilized laccase on the magnetic supports together with the fixed bed provided a promising avenue for the continuous enzymatic degradation of phenolic compounds.

## CONFLICT OF INTEREST

The authors have declared no conflict of interest.

## Supporting information


**Fig. S1**. Dynamic curves of phenol degradation by Fe_3_O_4_–NH_2_–PEI–laccase in the fixed bed and in shake flask.

## Data Availability

The data that support the findings of this study are available from the corresponding author upon reasonable request.
