# Supplementary material for: Efficient phenol degradation by laccase immobilized on functional magnetic nanoparticles in fixed bed reactor under high‐gradient magnetic field
Source: Eng Life Sci. 2021 May 6;21(6):374–81. doi: 10.1002/elsc.202100009 (PMC8182289; doi:10.1002/elsc.202100009)
Supplement: Supplementary file 1 — Fig. S1. Dynamic curves of phenol degradation by Fe3O4–NH2–PEI–laccase in the fixed bed and in shake flask. [file ELSC-21-374-s001.docx]

**Xia et al. Fig. S1**
